# Pulmonary surveillance in inborn errors of immunity: current challenges and emerging approaches

**DOI:** 10.3389/fimmu.2026.1797843

**Published:** 2026-05-20

**Authors:** Ernest Hoptioncann, Rachel L. Eddy, Catherine M. Biggs, Jonathan H. Rayment

**Affiliations:** 1 BC Children’s Hospital Research Institute, Vancouver, BC, Canada; 2 Department of Pediatrics, University of British Columbia, Vancouver, BC, Canada; 3 Centre for Heart Lung Innovation, St. Paul’s Hospital, Vancouver, BC, Canada

**Keywords:** computed tomography (CT), magnetic resonance imaging (MRI), multiple breath washout (MBW), pulmonary complications, pulmonary function tests (PFTs)

## Abstract

Inborn errors of immunity comprise a heterogeneous group of disorders characterized by impaired immune function and a high burden of pulmonary complications, which are major contributors to morbidity and mortality. Lung involvement arises primarily through recurrent infection leading to airway disease, or through immune dysregulation causing non-infectious interstitial lung disease. Effective management requires sensitive tools to detect and monitor diverse pulmonary manifestations across the lifespan. Although conventional approaches, including pulmonary function testing and radiologic imaging, remain valuable, they have important limitations in this population, such as limited sensitivity, reduced feasibility in children, and cumulative radiation exposure. Emerging techniques, including multiple-breath washout and pulmonary MRI, offer radiation-free, complementary assessments of lung function and structure with the potential to improve detection and longitudinal monitoring of pulmonary complications in individuals with inborn errors of immunity.

## 1 The prevalence and burden of pulmonary complications

Inborn errors of immunity (IEIs) are a heterogeneous group of disorders characterized by absent or dysfunctional components of the immune system that can predispose individuals to infection and immune dysregulation. Pulmonary complications are a major burden on the health of people living with IEIs (pwIEIs), due to their high prevalence and severity. Data from the United States Immunodeficiency Network, indicate that nearly 40% of pwIEIs develop pulmonary complications, most commonly affecting the airways (86.8%), with less frequent involvement of the parenchyma (18.5%), pleura (4.6%), and vasculature (4.3%). The prevalence and type of pulmonary complication varies according to the underlying IEI. For example, individuals with common variable immunodeficiency (CVID) account for nearly half of all reported pulmonary complications, highlighting the disproportionate pulmonary burden in certain IEI populations (1).

Other studies have identified pulmonary complications as a major cause of mortality in pwIEIs. Using U.S. mortality data from 1999-2014, Pérez and colleagues reported that pulmonary complications accounted for 17.4% of deaths in pwIEIs, only second to extrapulmonary infections (34.6%). Like prevalence, the contribution of pulmonary complications to mortality is closely related to IEI diagnosis. In CVID, up to 24% of deaths had a pulmonary cause, compared with only 11–14% in several innate immune defects (2). Analyses of specific IEIs, such as X-linked agammaglobulinemia (XLA), similarly show a substantial pulmonary burden, with pulmonary complications contributing to 41% of reported deaths (3).

Taken together, the high prevalence and severity of pulmonary complications in pwIEIs underscore the need for tools that enable disease detection, longitudinal monitoring, and assessment of treatment response.

## 2 How pulmonary complications develop

The causes of pulmonary complications in pwIEIs can be broadly categorized into two main underlying drivers: recurrent infection and non-infectious immune dysregulation.

### 2.1 Recurrent infection

Genetic defects in the immune system frequently impair fundamental components of host defense, including pathogen recognition, lymphocyte development, and effector function (4). These deficits compromise microbial clearance and immune homeostasis, predisposing individuals to recurrent or persistent infections; effects that are especially apparent in the respiratory tract due to its large surface area and continuous exposure to the external environment (5).

Among IEIs, humoral immunodeficiencies such as CVID and XLA, which disrupt B-cell maturation and functional antibody production, are particularly subject to recurrent respiratory infections (3, 6). Over time, these repeated infections trigger a self-perpetuating cycle of airway injury and immune-mediated inflammation, ultimately causing chronic airway disease, most notably bronchiectasis (7, 8).

This process starts with injury to airway epithelial cells which release pro-inflammatory cytokines and adhesion molecules, such as ICAM-1, to recruit and activate immune and mesenchymal cells (9). What follows is an acute inflammatory response, involving neutrophils, eosinophils, and fibroblasts, that drive cytokine production, extracellular matrix turnover and cytotoxic responses (10, 11). While essential for pathogen clearance, unresolved inflammation drives structural changes to the airway that impair mucociliary clearance, promote pathogenic colonization, and perpetuate a, self-reinforcing cycle of injury and inflammation (12). With time, the airways permanently become enlarged, distorted, or cystic, obstructing airflow and impairing lung function (13).

### 2.2 Non-infectious immune dysregulation

Immune dysregulation, characterized by an inability to control immune responses, is a feature of many IEIs, and underlies a range of clinical manifestations observed in these disorders such as inflammation, autoimmunity, lymphoproliferation and malignancy.

A representative example is Cytotoxic T-lymphocyte associated protein-4 (CTLA-4) haploinsufficiency, a condition which arises from the loss of one functional *CTLA-4* allele and reduced CTLA-4 surface expression. Diminished availability of this key immunoregulatory molecule impairs the downregulation of costimulatory signaling between T cells and antigen presenting cells, through the CD80/CD86–CD28 interaction. The resulting unchecked T-cell activation and expansion, then drives chronic lymphocytic infiltration and inflammation within the lungs (14, 15).

Unlike infectious pulmonary complications, which begin with airway epithelial injury, inflammatory pulmonary complications like granulomatous lymphocytic interstitial lung disease (GLILD) arise from immune dysregulation within the interstitial spaces of the lung. The initiating trigger is unknown, however thought to be antigenic material (microbial, environmental, or self-derived) (16). This in turn, drives activation of resident macrophages, which initiate granuloma formation and engage in signaling pathways such as mTOR, and JAK/STAT, promoting a chronic, non-infectious inflammatory response (17, 18). The adaptive immune response further amplifies this process, where the loss of regulatory control mechanisms, such as defective CTLA-4–mediated T-cell suppression (19), promote lymphoproliferation and the development of tertiary lymphoid structures within the lung parenchyma (18, 20). When persistent, this dysregulated immune activity causes irreversible damage characterized by interstitial fibrosis, leading to progressive loss of lung function. In particular, pathophysiological thickening of the delicate alveolar–capillary membrane leads to impaired gas exchange, and thickening of the interlobular septa leads to reduced respiratory system compliance (21, 22).

These mechanistic perspectives help explain why pulmonary complications are considered clinically distinct entities that affect specific anatomical regions with distinct functional consequences (23). Despite this, overlap can occur; Pneumocystis jirovecii pneumonia, for example, may present with diffuse interstitial changes that closely mimic non-infectious interstitial lung disease (24, 25), while cavitary lesions may arise from either chronic dysregulated inflammation or opportunistic infection, as seen with Nocardiosis (26, 27).

## 3 The importance of monitoring for pulmonary complications

Although pulmonary complications in pwIEIs are highly prevalent and often severe, several therapeutic approaches have shown meaningful benefits in restoring lung function and extending years of life. Combination immunosuppressive therapy has demonstrated efficacy at reducing radiographic abnormalities and improving pulmonary function in patients with GLILD (28). Similarly, intravenous immunoglobulin (IVIG) therapy, for chronic lung disease, has been shown to improve lung function, radiographic appearance (29), as well as longevity and quality of life (30).

Despite these therapeutic advances, optimal outcomes in pwIEIs ultimately rely on a simple principle: without screening, pulmonary complications remain undetected, and, consequently, cannot be treated. Pulmonary surveillance is therefore essential for several reasons. First, it enables early detection: radiographic screening can reveal preventable pulmonary changes in both children and adults with CVID (31, 32). Second, it enables disease characterization: imaging can identify specific pulmonary complications that may warrant further investigation, such as bronchoalveolar lavage or lung biopsy, to guide therapy (33–35). Third, it supports monitoring of treatment efficacy: regular lung function testing can assess whether interventions, such as IVIG, can maintain pulmonary stability (36, 37). In sum, sensitive and proactive surveillance is critical for detecting, defining, and effectively treating pulmonary complications in pwIEIs.

## 4 How pulmonary complications are characterized

The current clinical tools to assess lung health and monitor the progression of disease, fall into two general categories: pulmonary function tests (PFTs) and radiographic imaging.

### 4.1 Pulmonary function tests

PFTs are the most widely used method for monitoring lung function and are particularly important in the management of pwIEIs, as they are non-invasive, readily available across multiple levels of care, and provide quantitative assessment of multiple aspects of lung health (38). A longitudinal assessment of PF testing in 20 pwIEIs over an average of 101 months, using spirometry (FEV_1_) and diffusing capacity (DL_CO_), identified within-individual declines in FEV_1_ in 7 (35%), and reduced DL_CO_ in additional 5 (25%). Notably, two of these 12 (16.7%) had no radiographic abnormalities, underscoring the value of PFTs in occasionally identifying subclinical functional decline (37). Because of their clinical utility, PFTs are routinely performed in pwIEIs, even in the absence of overt lung disease. Real-world testing intervals vary across centers and are influenced by infection frequency, evidence of pulmonary involvement, and patient age (39). Published practices range from every 6 months (40) to every 1–3 years (41); however most guidance supports annual assessment as a practical minimum standard for both adults and children able to perform PFTs (typically >6 years) (42, 43).

While routine and clinically informative, PFTs lack the sensitivity to detect many early or subtle signs of lung disease that are often revealed by imaging. One study reported that fewer than one third of individuals with CVID and significant structural lung disease identified by imaging had detectable abnormalities on PF testing (32). Further, these tests are limited in pediatrics, as most children under the age of six struggle to provide the level of cooperation and effort needed to generate reliable and clinically useful results (44, 45). Overall, PFTs are valuable tools for pwIEIs because they are non-invasive, readily accessible and assess multiple aspects of lung function; however, are limited by poor sensitivity to mild disease and a lack of feasibility in young children.

### 4.2 Radiographic imaging

**Figure 1:**
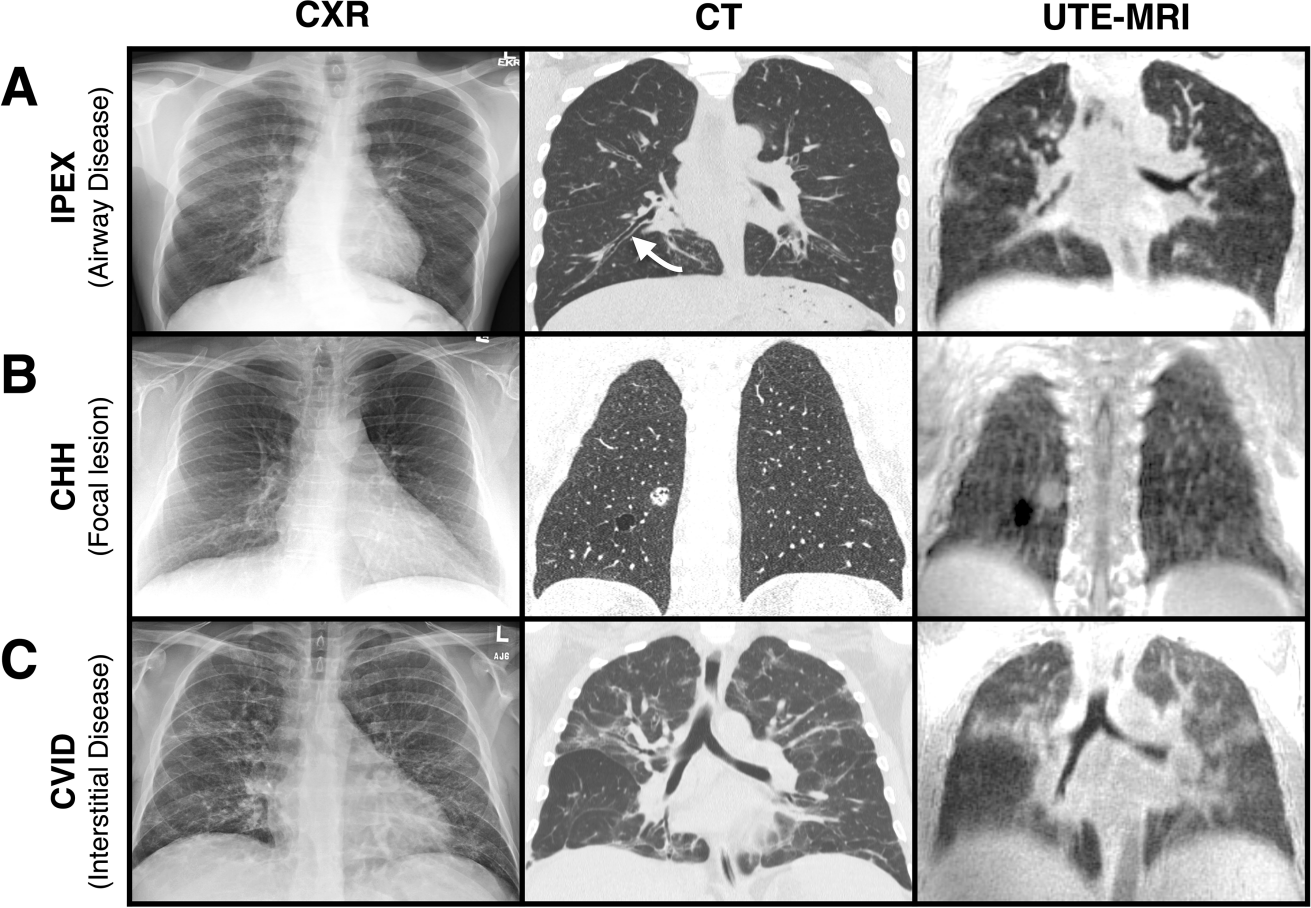
Radiologic manifestations of inborn errors of immunity across pulmonary imaging modalities. Radiologic manifestations in three distinct inborn errors of immunity (IEI) are shown across complementary pulmonary imaging modalities: chest radiograph (CXR), computed tomography (CT), and ultra-short echo time magnetic resonance imaging (UTE-MRI). Radiographic findings **(A)** Immunodysregulation polyendocrinopathy enteropathy X-linked (IPEX) syndrome: peripheral airway plugging and bronchiectasis in the posteromedial right lower lobe (indicated by white arrow); **(B)** cartilage-hair hypoplasia (CHH): a 22 × 18 mm part-solid, predominantly ground-glass and cystic nodule in the right lower lobe; **(C)** common variable immunodeficiency (CVID): stable bilateral peribronchovascular and peripheral reticulation, interstitial thickening, and architectural distortion.

Pulmonary imaging primarily relies on two radiographic techniques: chest X-rays (CXR) and computed tomography (CT). CXRs are widely available, quick to perform, and expose patients to a relatively low dose of radiation (~0.1 mSv, about 3-5% of annual background radiation) (46). As such, they are useful initial screening tools but provide only a two-dimensional projection (**Figure 1**), which limits their sensitivity for detecting subtle or early structural lung changes. In pwIEIs, important abnormalities such as interstitial changes and bronchiectasis are often missed on CXR. For example, in 54 pwIEIs, CT identified bronchiectasis in 15 scans (27.7%) and parenchymal nodules in 5 (9.2%). In contrast, CXR detected bronchiectasis in only 1 radiograph (3.7%) and failed to identify any nodules (47).

CT’s enhanced ability to evaluate lung architecture and detect signs of disease comes from its high-resolution 3D images, typically with submillimeter resolution (**Figure 1**). Currently, CT remains the reference standard for detailed pulmonary evaluation, offering the highest sensitivity for detecting structural airway or interstitial pathology (21, 48). A systematic review in CVID showed that detected abnormalities missed by CXR and PFT in 2–59% of cases (49), suggesting that undiagnosed lung disease could affect anywhere from 1 in 50 to as many as 30 in 50 individuals. As such, performing baseline CT is widely recommended in pwIEIs, typically at diagnosis and regardless of apparent lung disease. However, there is no universally accepted interval for repeat imaging; frequency varies across centers and is typically individualized based on symptoms, infection burden, and evidence of progression (39). In high-risk groups, such as those with GLILD, follow-up CTs may be obtained as frequently as every 3–5 months during treatment (50). In contrast, for asymptomatic adults with CVID, repeat imaging is generally performed every 2–5 years (41, 43). Despite these advantages, CT imaging carries a substantially higher radiation dose than CXR, approximately 50–100 times greater, with even low dose protocols exceeding CXR exposure by 4–12 times (51, 52). Consequently, in children CT is recommended primarily to monitor disease progression rather than for routine surveillance in asymptomatic patients (39). Further, in IEIs with DNA-repair defects, such as ataxia–telangiectasia or Nijmegen breakage syndrome, CT is generally avoided wherever possible (53), and may pose non-trivial risk in subsets of individuals with CVID who demonstrate chromosomal radiosensitivity *in vitro* (41, 54, 55). Overall, radiographic imaging, particularly CT, is essential in pwIEIs due to its superior structural resolution and sensitivity to regional disease; however, its longitudinal use is constrained by cumulative radiation exposure, particularly in children and individuals with radiosensitive IEIs.

## 5 New techniques to monitor pulmonary complications

As with the current diagnostic armamentarium, emerging techniques fall into two categories: functional testing and imaging. However, these novel approaches differ in their potential to address longstanding limitations of traditional methods particularly by improving diagnostic sensitivity and enhancing feasibility without the risk of radiation. Given the clinical and pathobiological heterogeneity of IEIs and their pulmonary manifestations, there remains a clear need for complementary tools that can capture distinct aspects of lung structure and function and thereby improve assessment of disease progression and treatment response (**Table 1**).

**Table 1:** Comparison of pulmonary surveillance modalities in inborn errors of immunity.

Modality	Primary assessment	Advantages	Disadvantages
PFTs Pulmonary function testing	Global lung function (airflow, lung volumes, gas exchange)	Widely available; low cost; standardized across centers	Insensitive to early/regional disease; limited feasibility in young children
MBW Multiple breath washout	Ventilation heterogeneity	Sensitive to early disease; feasible in children	Requires specialized expertise; limited standardization restricted to specialized centers
CXR Chest X-ray	2D structural imaging	Widely available; low cost	Insensitive to early disease; 2D projection limits structural detail
CT Computed tomography	3D structural imaging	High spatial resolution; Gold standard for structural airway and interstitial disease	Exposure to radiation, significantly higher than CXR; unsuitable for frequent longitudinal use, especially in children/radiosensitive IEIs
MRI Magnetic resonance imaging	3D structural imaging	Radiation-free 3D imaging; improving structural characterization (e.g. UTE/ZTE sequences)	Limited availability; high cost; technical complexity; lower spatial resolution than CT; limited standardization
XeMRI Hyperpolarized ^129^Xe MRI	3D functional imaging	Provides multi-dimensional assessment of lung function (ventilation, microstructure, gas exchange); Highly sensitive to early regional abnormalities	Very limited availability; high cost (MRI, polarizer, xenon gas); complex acquisition/analysis, requiring expertise restricted to specialized centers; no IEI specific data

### 5.1 Multiple breath washout

One potentially valuable functional test is the multiple breath washout (MBW) test (56, 57), which measures the washout of an inert tracer gas, such as sulfur hexafluoride (SF_6_) or nitrogen (N_2_), during tidal breathing through a mouthpiece or mask by breathing in room air or 100% oxygen (58). The primary outcome, known as the lung clearance index (LCI), indicates the cumulative exhaled volume (relative to the lung’s functional residual capacity) needed to wash out the tracer gas, a higher number indicating poorer lung function. In essence, it reflects how efficiently gas is mixed within the lungs, providing a sensitive measure of abnormal lung function, particularly that of the small airways (59).

In cystic fibrosis (CF), studies have shown that LCI detects structural airway disease, well before it is evident on standard tests like spirometry (60–62). Since the MBW test requires only tidal breathing, it is better suited for young children who may struggle with effort-dependent tests like spirometry (45), making it particularly useful for monitoring conditions such as CF, which can exhibit subclinical airway disease at an early age (63, 64).

To date, only a single study has investigated MBW in pwIEIs (65). In this study, MBW demonstrated greater sensitivity than spirometry in identifying functional abnormality: 35.1% (40 of 114) exceeded the abnormal threshold for LCI, compared with 20.7% (19 of 92) for FEV_1_. Furthermore, 20.7% of individuals with an abnormal LCI had a normal FEV_1_, indicating the detection of subclinical disease not captured by spirometry. Finally, over a median interval of 364 days, LCI increased significantly by 0.2, in 70 individuals, indicating that LCI is sensitive to short-term disease progression, even in clinically stable individuals. Overall, these findings support MBW as a sensitive tool for detecting early pulmonary dysfunction and monitoring disease progression in IEIs. However, they are limited by a lack of prospective and long-term follow-up data, necessitating further evidence to define its clinical role.

### 5.2 Magnetic resonance imaging

Given the increased radiosensitivity and elevated cancer risk in pwIEIs (66), there is a clear need to minimize exposure to ionizing radiation through non-radiographic approaches to imaging, such as magnetic resonance imaging (MRI).

MRI signal depends on the presence of hydrogen nuclei, which are highly abundant in most biological tissue. However, the lung is primarily composed of air (~80% of its volume), offering relatively few nuclei to generate an image (67). This low intrinsic signal, compounded by challenges like air-tissue interfaces and physiological motion, has historically limited MRI’s capacity to produce high-resolution images suitable for clinical pulmonary evaluation when compared to CT (68, 69). Despite this limitation, the inherent advantage of radiation-free imaging has fueled considerable interest and technological advancement in pulmonary MRI.

Studies comparing structural MRI with CT for identifying pulmonary complications in pwIEIs demonstrate that MRI provides sufficient resolution to detect most airway and parenchymal abnormalities, such as large-airway bronchiectasis and consolidation (70–72). However, MRI is less effective at assessing the full anatomical extent of bronchiectasis and the depth of airway involvement. Across all three studies, MRI showed poor agreement with CT for both measures, particularly in mild disease found in the small airways. For example, CT detected bronchiectasis beyond the sixth bronchial division (peripheral airways) in 67–83% of cases, compared with 38–61% by MRI.

These shortcomings reflect the current spatial and contrast resolution limits of conventional pulmonary MRI. However, rapid developments in ultrashort-echo time (UTE) (**Figure 1**) and zero-echo time (ZTE) sequences are improving image quality and the characterization of airway and parenchymal disease. Studies of lung neoplasms show that ZTE-MRI provides good visualization of subsegmental bronchi, and near-excellent lesion detectability, approaching the performance of CT (73, 74). Further, synthetic CT images generated from UTE-MRI in individuals with CF showed near-perfect agreement with real CT for assessing the extent of bronchiectasis and peripheral mucus plugging, areas of previous limitation (75). Collectively, these advances suggest that novel approaches in MRI are beginning to approach CT-level sensitivity, offering a potential radiation-free alternative for longitudinal lung imaging.

MRI can also yield physiologic insights, when paired with novel technologies. Exogenous MR-visible nuclei, such as ^129^Xe, which can undergo a process called “hyperpolarization” outside the MRI scanner to dramatically enhance signal (76). When inhaled, ^129^Xe distributes throughout the lung providing detailed functional information on ventilation, microstructure, and (due to its partial solubility in tissues and the blood)-gas exchange (77).

To date, hyperpolarized ^129^Xe MRI (XeMRI) has never been investigated in pwIEIs. However, it may represent a promising approach, given that pulmonary complications in IEIs encompass both airway and interstitial disease. XeMRI has demonstrated sensitivity to airway abnormalities beyond conventional PFTs in CF and asthma (78, 79), while also revealing gas-exchange impairment in fibrotic ILDs (80–82). In short, these capabilities position XeMRI as a sensitive tool for regional lung function in IEIs with potential for earlier detection and more comprehensive pulmonary assessment.

Still, as with other highly sensitive imaging modalities, uncertainty remains regarding the potential for overdiagnosis, as observed in CT-based cancer screening, where detection of benign abnormalities has led to unnecessary interventions in otherwise stable individuals (83). A key challenge will be balancing increased diagnostic sensitivity with appropriate clinical interpretation to avoid unintended harm.

## 6 Conclusions

Overall, pulmonary complications are a major driver of morbidity and mortality in pwIEIs and therefore require effective monitoring strategies to guide treatment. While current monitoring tools are clinically invaluable, each has limitations: PFTs lack sensitivity to mild disease and are often infeasible in young children, whereas radiographic imaging relies on ionizing radiation, limiting its suitability for longitudinal monitoring. Emerging approaches offer promise: MBW can improve sensitivity to early physiological impairment in both adults and children, and MRI provides radiation free structural and functional assessment. While further prospective work is required in this population, these techniques offer complementary insights into lung health and highlight the potential to enhance early detection and reshape monitoring strategies in pwIEIs.
